# Management of an extensive odontogenic keratocyst: A rare case report with 10-year follow-up

**DOI:** 10.1097/MD.0000000000017987

**Published:** 2019-12-20

**Authors:** Abdul Ahad Khan, Saad Al Qahtani, Ali Azhar Dawasaz, Shahabe A. Saquib, Shaik Mohammed Asif, Mohammad Ishfaq, Mohammad Zahir Kota, Mohammed Ibrahim

**Affiliations:** a Department of Oral and Maxillofacial Surgery, College of Dentistry; b Department of Periodontics and Community Dentistry; c Department of Diagnostic Sciences and Oral Biology; d Department of Oral and Maxillofacial Surgery, College of Dentistry, King Khalid University, Abha, Saudi Arabia.

**Keywords:** bcl-2, keratocystic odontogenic tumor, Ki-67, odontogenic keratocyst, panmandibular

## Abstract

**Introduction:**

The odontogenic keratocyst (OKC), previously known as keratocystic odontogenic tumor has been the most disputable pathologies of the maxillofacial region. Patients with OKC are often asymptomatic but may present with pain, swelling, or discharge. Despite the aggressive nature, previous literature as early as 1970s reported the fact that parakeratinized OKC can be treated by means of marsupialization alone.

**Patients concerns:**

The patient had reported with a complaint of pain and swelling in relation with a tooth in mandibular right quadrant.

**Diagnosis:**

This case report discusses features of a rare, extensive, panmandibular OKC that is only second of its kind mentioned in the literature.

**Intervention:**

As a usual treatment protocol, marsupialization was attempted first. Immunohistochemical analysis revealed reduced expression of Ki-67 and B cell lymphoma 2 (bcl-2) markers after marsupialization from 2 separate sites. However, due to incomplete resolution in the lower right anterior region, an aggressive approach was taken by curetting it out surgically along with associated teeth and cortical plate followed by application of Carnoy's solution.

**Outcome:**

Postsurgery uneventful healing of the lesion was noted on regular follow-up visits with complete resolution at 40 months. The case has been followed for 10 years with no sign of relapse and reoccurrence.

**Conclusions:**

Based on the expression of markers it can thus be concluded that Ki-67 and bcl-2 are site specific and bear strong relationship with the recurrence of OKCs.

## 1 Introduction

The odontogenic keratocyst (OKC) has been a standout amongst the most disputable pathological entities of the maxillofacial region since the mid of 20th century.^[1]^ The OKC is known for its ambiguity since its inception. Initially it was mistaken for a primordial cyst. Later its aggressive clinical behavior and tendency for recurrence literally detached its association from the category of cysts and it came to be known as an intraosseous benign neoplasm, keratocystic odontogenic tumor (KCOT) (World Health Organization [WHO] classification 2005).^[2]^ Ironically, there were reports of OKC responding to marsupialization and hence the revised classification by WHO, published in 2017,^[3]^ reclassified the OKC as a cystic lesion instead of benign intraosseous neoplasm, KCOT.^[2]^ In spite of extensive research, we have still not arrived at a consensus as to the behavior of this obscure entity. Approximately one-half of all keratocysts occurs at the angle of the mandible and extend for varying distances into the ascending ramus and forward into the body. In many instances, patients are remarkably free of symptoms until the cysts reached a large size and involved the maxillary sinus and the entire ascending ramus, including the condylar and coronoid processes.^[4]^

It's typical histological highlights incorporate a thin parakeratinized stratified squamous epithelium, roughly 5 to 8 cells thick, secured by a thin ridged layer of parakeratin^[5]^ characteristic palisaded pattern with uniform nuclei are seen in the basal cell layer.^[6]^ An important feature of OKCs being the daughter cysts which are formed by budding of basal layer into the surrounding connective tissue.^[7]^ The fibrous cyst wall is relatively thin and usually lacks inflammatory cell infiltrate.^[6]^

There has been reported recurrences ranging from 0% to 100%.^[8]^ These marked differences are believed to be identified with the distinctive duration of postoperative follow-up periods, surgical procedures utilized, or incorporation of cases with nevoid basal cell carcinoma disorder (NBCCS).^[9]^

Treatment strategies have been devised on the basis of molecular studies and extensive reviews of literature suggesting the modalities with reduced possibility of recurrence. Blanas et al^[10]^ have found a recurrence rate of around 17% to 56% when treated by simple enucleation in their systematic review of 14 investigations. They have also suggested that addition of Carnoy's solution to the cystic cavity for 3 minutes after enucleation. This reduced the recurrence to 1.6% which is comparable to resection, without associated morbidity.

Stoelinga^[11]^ has also proposed a treatment strategy based on the pattern of behavior of OKC. He has suggested careful cystic enucleation with excision of overlying mucosa and recommended electrocoagulation/Carnoy's solution application in selective areas where the cyst was attached to the soft tissues. On the other hand, marsupialization alone as the sole treatment modality for OKC was suggested by Pogrel^[12]^ with an average of 2.9 years follow-up. He even found uprighting and eruption of teeth in the cyst. His explanation to this was Immunohistochemistry evidence of higher interleukin-1 alpha levels in OKC that significantly reduced after marsupialization.^[13]^ In all his cases, histologic material taken after marsupialization showed normal epithelium with no daughter cysts or remnants or budding of basal epithelial layer. He found preoperative bcl-2 protein expression strictly limited to basal layer and postoperative bcl-2 negative normal oral mucosa specimen.^[12]^

To the best of authors’ knowledge, this rare extensive nature of lesion has been reported only once in the literature by Gupta et al.^[14]^ The line of management in our case was influenced by the classic work of Pogrel^[12]^ who had demonstrated that marsupialization can be considered as a definitive treatment modality for OKCs.

## 2 Case report

**Figure 1:**
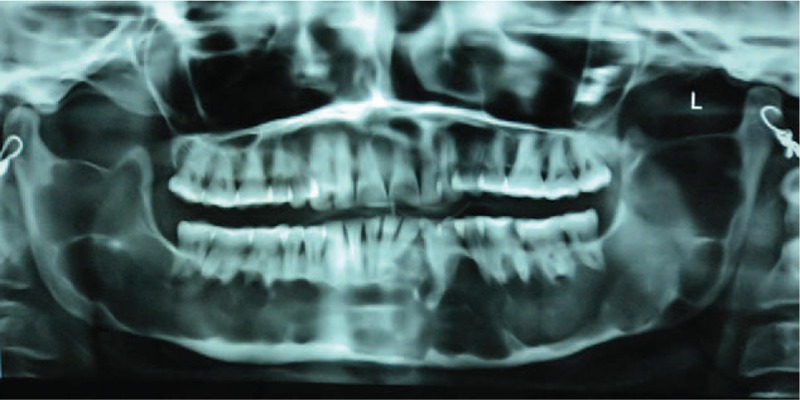
Preoperative radiograph showing an extensive radiolucent lesion spanning across the whole length of the mandible from one condyle to the other.

A 35-year-old female visited the Department of Oral and Maxillofacial Surgery with the complaint of pain on chewing and swelling on the right side of the face. Medical history was not contributory. On intraoral examination, the only positive finding was a grossly carious, tender, right mandibular first molar. Radiographic examination revealed an extensive, multilocular radiolucency bounded by a radiopaque (sclerotic) margin all around. Figure 1 shows radiolucency extended across the whole body of mandible, bilateral ramus without involvement of both the condyles. After thorough examination of the case the patient was diagnosed as having an infection involving the right buccal and submandibular space, secondary to a carious right mandibular first molar, along with a panmandibular cystic lesion, most probably an OKC. A provisional diagnosis was made as OKC due to presence of keratin on aspiration. The submandibular and buccal space infections were treated surgically and the offending tooth was extracted. Incisional biopsy was taken from the extraction wound and the diagnosis was confirmed as nonsyndromic parakeratinized OKC.

Marsupialization was considered as an appropriate treatment plan, as the lesion was quite extensive. So, bone windows were made by excising mucoperiosteum along with the bone in the left mandibular buccal vestibule and anterior mandibular labial vestibule. Since the extraction socket of mandibular right first molar provided a potential means of irrigation of the cystic cavity, it was enlarged by removal of interradicular bone by means of bone rongeur and rotary instruments with copious saline irrigation. The cystic contents were evacuated, and the cystic cavity was packed with tape gauze soaked (and squeezed) in 2% povidone-iodine for 3 days. This was followed by periodic irrigation and suction of the cystic cavity with 2% povidone-iodine and normal saline (1:1 proportion). The irrigation of the cystic cavity was initially done every alternate day for 15 days, then twice weekly for about 4 months followed by weekly irrigation. Acrylic plugs were prepared to prevent premature closure of the bony defects. Orthopantomograms were taken at regular intervals to monitor the progress. Histopathological examination was carried out from the base of the resolved lesion in the anterior mandibular area. The pretreatment biopsy specimen and the lesional tissue from the base of the resolved lesions were subjected to immunohistochemical analysis to assess the expression of Ki-67, a proliferative marker, and bcl-2, an antiapoptotic marker (BioGenex reagent and Super Sensitive polymer horseradish peroxidase kit Sigma Aldrich, Germany).

**Figure 2:**
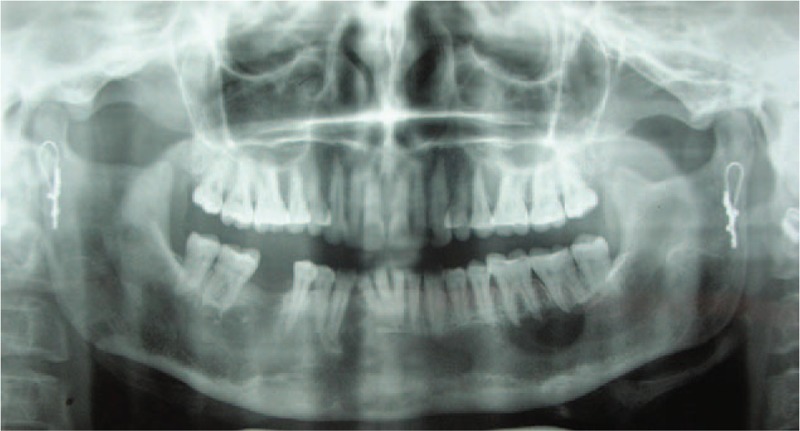
Radiograph at 2-year follow-up showing reduction in the radiolucency of the lesion from the periphery, except for the region between lower right lateral incisor and canine.

**Figure 3:**
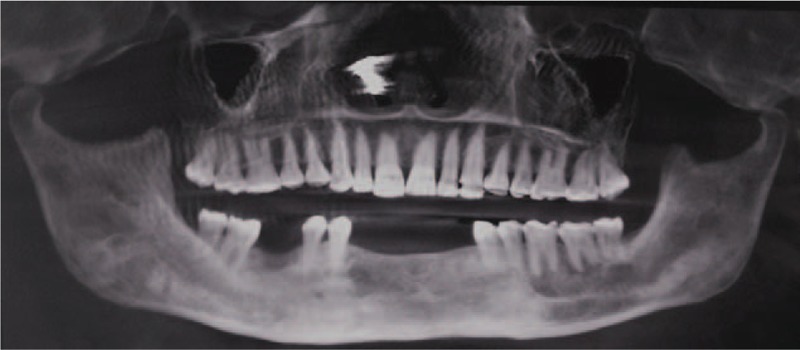
Postoperative radiograph at a 10-year follow-up showing complete resolution of the lesion as evidenced by replacement of radiolucency by radiopaque bone.

The preoperative radiograph revealed that the cystic lesion involved whole of the mandible, sparing both the condyles. Progressive reduction in the radiolucency was noted in successive radiographs taken. The radiographs taken at an interval of 4 months after marsupialization depicted gradual resolution of the lesion (as evidenced by a radiographic reduction in the radiolucency accompanied simultaneously by replacement with radiopaque bone), except for a small area in the mandibular right quadrant (4th quadrant). Clinically the bone windows have largely filled up from within, leaving a 1.5 cm depth, self-cleansing bony defect in the mandibular left quadrant, and a 0.5 cm self-cleansing, bony defect in the mandibular anterior area. The immunohistochemical report of the pretreatment biopsy specimen from lower anterior and lower left tissues showed the characteristic parakeratinized epithelium of OKC (Fig. 2) with an abundance of Ki-67 and bcl-2 in the basal and suprabasal layers. Whereas the tissue specimen from the base of the resolved lesion depicted the transformation of the classic OKC epithelium to stratified squamous epithelium (Fig. 3) with reduced density and expression of both Ki-67 and bcl-2

**Figure 4:**
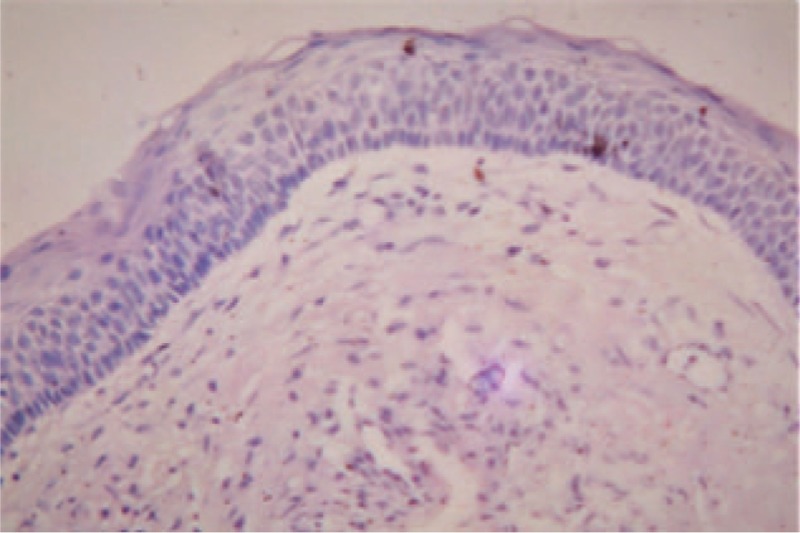
Preoperative histopathological picture showing the typical histopathologic picture of OKC.

**Figure 5:**
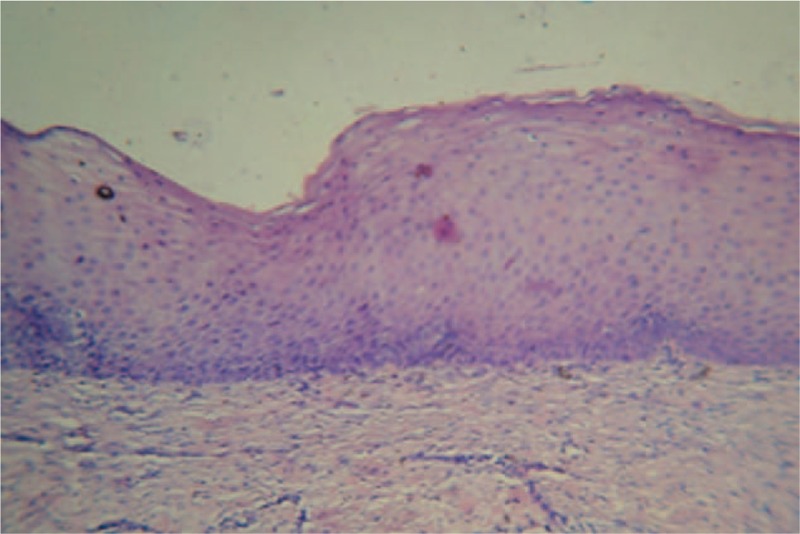
Posttreatment photograph showing metaplastic transformation of the classic OKC epithelium to stratified squamous epithelium of the oral mucosa.

The timeline of patient follow-ups have been summarized in Table 1. It was observed that there was reduction in overall radiolucency after a period of 2 years except in 42 and 43 region where root divergence was observed. (Fig. 4) So the area was curetted out and chemical cauterization was done with Carnoy's solution under local anesthesia. After 6 months of observation, there was no resolution of the radiolucency. So, an aggressive approach was taken by removing complete labial cortex along with associated teeth and a repeat chemical cauterization with Carnoy's solution. At 3-year follow-up, clinical loss of labial vestibule was observed. The patient then underwent prosthetic rehabilitation using tooth supported partial denture. Regular follow-up up to 10 years (Fig. 5) revealed total uneventful bone healing with no evidence of recurrence or new lesion.

**Table 1:**
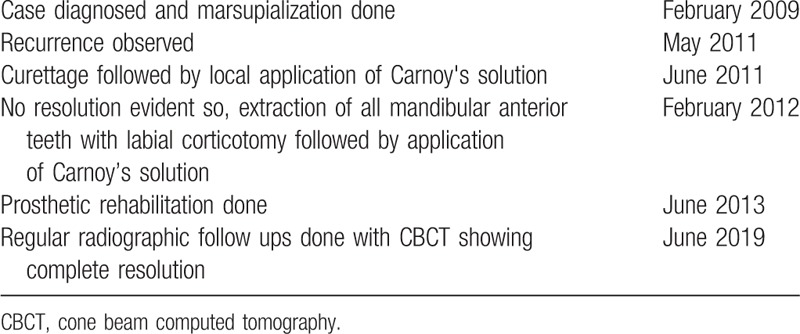
Timeline for the continued follow-up of the case.

## 3 Discussion

Based on extensive research, recommended treatment modalities for OKC that are known to reduce/prevent recurrence include enucleation, excision of overlying mucosa followed by application of Carnoy's solution,^[15]^ marsupialization^[16]^/decompression followed by cystectomy,^[17]^ and mandibular resection.^[18]^ However, in our case enucleation had the possibility of pathologic jaw fracture, whereas cystectomy and resection had their own share of morbidity including functional, psychologic, cosmetic, and financial implications. Marsupialization as a potential treatment modality for parakeratinized OKC has received a great deal of attention after the research work of Pogrel^[12]^; however, the findings of this case suggests otherwise.

The reduced expression of Ki-67 after marsupialization indicate the reduced proliferative activity and potential for recurrence and that of bcl-2 indicates the conversion of the classic OKC epithelium to the stratified squamous epithelium. The bcl-2 gene, located at chromosome 18q21, is characterized by its ability to stop apoptosis (programmed cell death) without promoting cell proliferation.^[19]^ Bcl-2 inhibits apoptosis to facilitate cellular proliferation in the basal and suprabasal layers, whereas apoptosis maintains the homeostasis of the thickness of the lining epithelium and allows the synthesis of large amounts of keratin in the surface layer of OKCs. There is a regulated balance between cell proliferation, cell differentiation, and cell death in this type of lesion, this may explain why OKCs do not transform into tumor masses, instead of having neoplastic behavior with an increased potential to proliferate.^[19]^

Ki-67 antigen is more specific marker of proliferating cells, maximally expressed during S-phase. The levels of Ki-67 in the epithelial linings were found to be double in syndrome-related OKCs as compared to the sporadic cysts, indicating a greater level of proliferative activity in the former. This was reflected in the multiplicity of cysts and the increased numbers of satellite cysts and epithelial islands in the OKCs of NBCCS patients. This suggested that it was a genetic factor, possibly related to defective tumor suppression functions, that was reflected by the higher proliferative activity in their epithelial linings.^[20]^ It was also observed that Ki-67 expression was found to be more common in odontogenic cysts and tumors that are more aggressive in nature.^[21]^

The study done by Ninomiya et al,^[13]^ revealed strong expression of interleukin (IL)-1α, mRNA and protein in the epithelial cells of OKCs, those reduced significantly after marsupialization. In fact, Ki-67 labeling index of the epithelial cells diminishes proportionally with the grade of IL-1α, mRNA expression after the marsupialization. This finding suggested that, marsupialization may reduce the size of OKCs by inhibiting IL-1α expression and the epithelial cell proliferation.^[20]^ It was observed in this case that there was significant reduction in Ki-67 and Bcl-2 at the healing sites.

At a 2-year follow-up radiolucency along with root divergence in 42 and 43 region was observed which was curetted out and chemical cauterization done. Six months post-operation, showed nonresolution of lesion that prompted the surgeons to adopt an aggressive approach by removing total labial cortex along with associated teeth. It was also observed that there was no reduction of Ki-67 and Bcl-2 from the above site where the lesion did not resolve by marsupialization alone. Our results counter the treatment plan of Gupta et al,^[14]^ in that extraction of all the mandibular teeth including third molars was done and full bilateral buccal decortication and cystotomy was performed for the panmandibular OKC.

In an attempt to cover the large denuded lingual cortical plate, we mobilized the tissue from the labial mucosa. However, this resulted in the loss of vestibule in the lower anterior region. Rehabilitation was done with a tooth supported prosthesis. After a period of 10 years, it was observed that the lesion had complete healing with no reported evidence of any recurrence or new lesion.

## 4 Conclusions

To conclude, the authors state that marsupialization must be considered as the first line of management, especially for an OKC of such an extensive size, but cannot be considered as a definitive treatment modality for OKC. Nonetheless, other aggressive surgical treatments like removal of cortical plate along with overlying mucoperiosteum should be considered when the lesions are nonresponsive. Our study also reinforces the paradox that OKC, reclassified by WHO^[3]^ in 2017 may represent true cystic tumors but they respond to simple treatment such as marsupialization.

## Acknowledgment

We would like to thank Dr Priti and Dr Atul Deshmukh for assistance in the Histopathologic diagnosis of the case. The authors extend their appreciation to the Deanship of Scientific Research at King Khalid University for funding this work through General Research Project under grant number (GRP-184-40).

## Author contributions

Abdul Ahad Khan orcid: 0000-0001-7078-2069.

AAK, SAQ has been involved in drafting and writing the manuscript. AAK, AAD made substantial contributions for the conception of the manuscript. AAK, SSA made substantial contributions for acquisition of clinical data. MIb and MZ has been involved in revising the manuscript critically for important intellectual content. SMA made substantial contributions for acquisition of radiological data. MIs made substantial contributions for acquisition of clinical data, for the conception of the manuscript and gave final approval for the version of the manuscript to be published. Each author has participated sufficiently in the work to take public responsibility for appropriate portions of the content, and agreed to be accountable for all aspects of the work in ensuring that questions related to the accuracy or integrity of any part of the work are appropriately investigated and resolved. All the authors have made significant contributions to the manuscript and have also read and approved the manuscript.

## Correction

Funding information has been added the acknowledgement section.
